# Pharmacological Studies of Tentacle Extract from the Jellyfish *Cyanea capillata* in Isolated Rat Aorta

**DOI:** 10.3390/md11093335

**Published:** 2013-08-30

**Authors:** Beilei Wang, Bo Zhang, Qianqian Wang, Zhi Zhang, Fei Nie, Guoyan Liu, Jiemin Zheng, Liang Xiao, Liming Zhang

**Affiliations:** 1 Department of Marine Biotechnology, Faculty of Naval Medicine, Second Military Medical University, Shanghai 200433, China; E-Mails: lilly_wang@126.com (B.W.); zhangbo_0824@163.com (B.Z.); abc_w@163.com (Q.W.); niefei527@126.com (F.N.); lgy_laurie@yahoo.com.cn (G.L.); trados1@126.com (J.Z.); 2 Emergency Department, Navy General Hospital of PLA, Beijing 100048, China; E-Mail: zzsmmu@126.com

**Keywords:** jellyfish, *Cyanea capillata*, tentacle extract, vasoconstriction, contractile response

## Abstract

Our previous studies demonstrated that tentacle extract (TE) from the jellyfish, *Cyanea capillata*, could cause a dose-dependent increase of systolic blood pressure, which seemed to be the result of direct constriction of vascular smooth muscle (VSM). The aim of this study is to investigate whether TE could induce vasoconstriction *in vitro* and to explore its potential mechanism. Using isolated aorta rings, a direct contractile response of TE was verified, which showed that TE could induce concentration-dependent contractile responses in both endothelium-intact and -denuded aortas. Interestingly, the amplitude of contraction in the endothelium-denuded aorta was much stronger than that in the endothelium-intact one, implying that TE might also bring a weak functional relaxation in addition to vasoconstriction. Further drug intervention experiments indicated that the functional vasodilation might be mediated by nitric oxide, and that TE-induced vasoconstriction could be attributed to calcium influx via voltage-operated calcium channels (VOCCs) from the extracellular space, as well as sarcoplasmic reticulum (SR) Ca^2+^ release via the inositol 1,4,5-trisphosphate receptor (IP_3_R), leading to an increase in [Ca^2+^]_c_, instead of activation of the PLC/DAG/PKC pathway or the sympathetic nerve system.

## 1. Introduction

In recent years, there has been growing evidence that jellyfish blooms are increasing globally in frequency and persisting longer than usual in response to anthropogenic disturbance and climate change [1,2]. With larger human population numbers and their need for recreation, the contact between humans and jellyfish is increasing. Correspondingly, the incidence of jellyfish stings is on the rise [3].

Jellyfish envenomation is often accompanied with local reactions, such as painful, linear, red, hive-like lesions. Occasionally, however, the sting results in systemic reactions, such as shock, respiratory failure, cardiovascular collapse and, even, death [3]. Since cardiovascular complications are the most severe and life-threatening events in jellyfish stings [4], many researches focused on the cardiovascular toxicity of jellyfish venom, developing specific prevention or therapy strategies [5]. However, our current understandings of the cardiovascular toxicity of jellyfish venom are very limited, and the underlying mechanism of cardiovascular abnormalities following jellyfish envenomation is still unclear [4,6]. The study on the cardiovascular toxicity of jellyfish venom is mainly hindered by the following reasons: (1) jellyfish venoms are fragile, thermolabile and liable to adhesion, which greatly hampers the identification and isolation of individual components [7,8]; and (2) the collection of venom samples is difficult, due to the small amount of the venoms in nematocysts [6]. To solve the problem, we have previously compared nematocyst venom with tentacle extract (TE, devoid of nematocyst) from the jellyfish, *Cyanea capillata*. Our result suggested that TE may serve as a potential alternative to the nematocyst venom for isolating and purifying cardiovascular toxicity proteins, because they share similar cardiovascular activity *in vivo* [6]. Besides, further *in vitro* studies verified that TE also possesses obvious cardiac toxicity [9]. However, so far, it remains unclear whether TE has direct vascular activity.

In our previous studies, we found that TE could cause a dose-dependent increase of systolic blood pressure in the first few hours, followed by a depressor effect in conscious rats. Considering the direct cardiac toxicity of TE, we hypothesized that TE-induced pressor response appeared to be the result of direct constriction of vascular smooth muscle (VSM), and the depressor phase might reflect a direct cardiomyotoxicity of TE or an indirect cardiac toxicity, due to coronary vasospasm [10]. However, currently, many research groups are focusing their study on the isolation and purification of cardiotoxic protein from jellyfish venom [11,12,13], so little attention has been paid to its vasoconstriction effect. In fact, an increasing number of clinical reports indicated that the patients stung by jellyfish were often accompanied with vascular spasm [3], and in some cases, serious complications appeared, such as brachial artery spasm [14], deep vein thrombosis [15], renal failure [3,16] and cerebrovascular events [17]. Therefore, it is important to explore the vasoconstrictive responses by jellyfish venom, as well as the underlying contractile mechanism, which may improve our knowledge of jellyfish venom’s mode of action and the development of more effective remedies against jellyfish envenomation.

Thus, this study aims to elucidate TE-induced vascular effects using rat isolated aorta. Since Ca^2+^ signaling is vital to excitation-contraction coupling (E-CC) of vascular smooth muscle cells (VSMC) [18] and endothelium-derived factors [19] and the sympathetic nerve system [20] play important roles in regulating VSMC contraction, we also attempt to investigate if the blockers of these pathways would alter TE-elicited vasoconstrictions, for exploring the possible vasoconstrictor mechanism and potential therapeutic or prophylactic agents.

## 2. Materials and Methods

### 2.1. Animal Handling and Ethics Statement

Male Sprague-Dawley (SD) rats (280 ± 20 g) were provided by the Laboratory Animal Center of the Second Military Medical University. All the animals were housed under standard laboratory conditions with a 12/12 h light/dark cycle at 22 ± 2 °C and given standard diets plus water *ad libitum*. The investigation was carried out in conformity with the requirements of the Ethics Committee of the Second Military Medical University and international guidelines for animal handling. Jellyfish catching was permitted by the East China Sea Branch, State Oceanic Administration, China.

### 2.2. TE Preparation from the Jellyfish, *C. capillata*

Specimens of *C. capillata* were collected in June, 2011, in the Sanmen Bay, East China Sea, and identified by Professor Huixin Hong from the Fisheries College of Jimei University, Xiamen, China. The removed tentacles were preserved in plastic bags on dry ice and immediately shipped to Shanghai, where the samples were frozen at −70 °C until use. The TE was prepared following the method as described in previous reports [6,9]. Briefly, frozen tentacles were thawed at 4 °C and immersed in filtered seawater at a mass/volume ratio of 1:1 to allow autolysis of the tissues for four days. The mixture was stirred for 30 min twice daily. The autolyzed mixture was centrifuged at 10,000× *g* for 15 min, thrice. The resultant supernatant was the TE. All procedures were performed at 4 °C or in an ice bath. The TE was centrifuged at 10,000× *g* for 15 min to remove the sediments, followed by dialysis against phosphate buffered saline (PBS, 0.01 mol/L, pH 7.4) for over 8 h before use. The protein concentration in the preparations was determined using the method of Bradford.

### 2.3. Preparation of Aortic Rings for Tension Measurement

Male SD rats were anesthetized with urethane (1.0 g/kg i.p.). Then, the descending thoracic aorta was rapidly dissected out and placed into a petri dish with cold modified Krebs-Henseleit solution containing (in mM): NaCl (119), KCl (4.74), KH_2_PO_4_ (1.18), MgSO_4_ (1.18), NaHCO_3_ (24.9), CaCl_2_ (2.5) and glucose (12.0). The aorta was carefully cleaned of blood, fat and connective tissue and cut into 3-mm-long ring segments, which were: (1) suspended horizontally and (2) bathed in individual 10-mL tissue chambers filled with Krebs-Henseleit solution at 37 °C and bubbled with 95% O_2_ and 5% CO_2_ (pH 7.4) [21]. Changes in tension were recorded by isometric transducers connected to a data acquisition system (ALC_B10_, MPA-2000, Alcott Biotech, China). The rings were stretched, until a resting tension of 1 g was reached, and allowed to equilibrate for at least 45 min, during which time, tension was adjusted, as necessary, to 1 g. The bathing solution was periodically changed. Before each experiment, rings were stimulated with high potassium solution (KCl, 60 mM), until the responses were reproducible.

In some experiments, endothelium was removed by gentle rubbing of the luminal side with a 40-μm stainless steel wire. The integrity of endothelium was checked by the addition of acetylcholine (ACh, 10 μM) to phenylephrine (PE, 1 μM) pre-contracted preparations. The endothelium was considered intact if stimulation with ACh produced ≥60% relaxation. The endothelium was considered denuded if stimulation with ACh produced ≤10% relaxation [22].

### 2.4. Experimental Protocol in Isolated Aortic Rings

In the first set of experiments, an attempt was made to verify TE-induced contractile response. We added various doses of TE (1.5–72 µg/mL) in both endothelium-intact and -denuded rings. Contractions were expressed as percentages of maximum KCl contraction. Then, an appropriate concentration of TE (18 μg/mL) was chosen to examine the vasoactive effects of TE on PE-induced contraction in both endothelium-intact and -denuded rings.

The second series of experiments investigated the involvement of the endothelium-derived vasodilators (NO and prostacyclin) in the TE-induced contraction. Nitric oxide synthase (NOS) inhibitor, *N*^ω^-nitro-l-arginine methyl ester (l-NAME, 1 μM), and cyclooxygenase inhibitor, indomethacin (1 μM), were incubated 20 min before the stimulation of TE in the endothelium-intact rings.

The third series explored the possible contractile mechanism of VSM. Firstly, we examined the dependence of TE-induced contraction on extracellular calcium. Endothelium-denuded aortic rings were incubated in standard Krebs solution, low-calcium Krebs solution (1 mM CaCl_2_) and calcium-free Krebs solution containing ethylene glycol tetraacetic acid (EGTA, 1 mM) for 20 min before the addition of TE, respectively. Secondly, to confirm the participation of voltage-operated calcium channels (VOCCs) in VSMC contraction, the rings were pre-incubated 20 min with different types of VOCC blockers, including nifedipine (1 μM), diltiazem (1 μM) and verapamil (1 μM). Then, to characterize the possible contribution of the release of Ca^2+^ from sarcoplasmic reticulum (SR), the rings were pre-incubated with phospholipase C inhibitor (U-73122, 10 μM), protein kinase C inhibitor (PKC 412, 5 μM) and inositol 1,4,5-trisphosphate (IP_3_) receptor blockers: 2-aminoethoxydiphenyl borate (2-APB, 10 μM) and heparin (2500 IU). Finally, to examine the possible participation of the sympathetic nerve system, the rings were pre-incubated with propranolol (1 μM), a non-selective and competitive β-adrenergic receptor blocker, and phentolamine (1 μM), a non-selective α-adrenergic receptor antagonist. All the inhibitors (Sigma-Aldrich, Shanghai, China) whose effective concentrations were screened out in the preliminary experiment in similar preparations were incubated in the endothelium-denuded aortic rings, for at least 20 min, before the addition of TE.

### 2.5. Statistical Analysis

All data are expressed as the mean ± SD. The sigmoidal concentration-contraction response curves were fitted using GraphPad Prism 5 (GraphPad Software, San Diego, CA, USA) for each individual experiment. The EC_50_ (the concentration (μg/mL) required to produce 50% maximum response) and maximum responses (*E*_max_) of each treatment group were compared to the control (No drug) using one-way analysis of variance (ANOVA), with Dunnett’s *post hoc* test (GraphPad Prism 5). A *p*-value of less than 0.05 was considered statistically significant.

## 3. Results

### 3.1. Contractile Response Induced by TE in the Endothelium-Intact and Endothelium-Denuded Aorta

**Figure 1:**
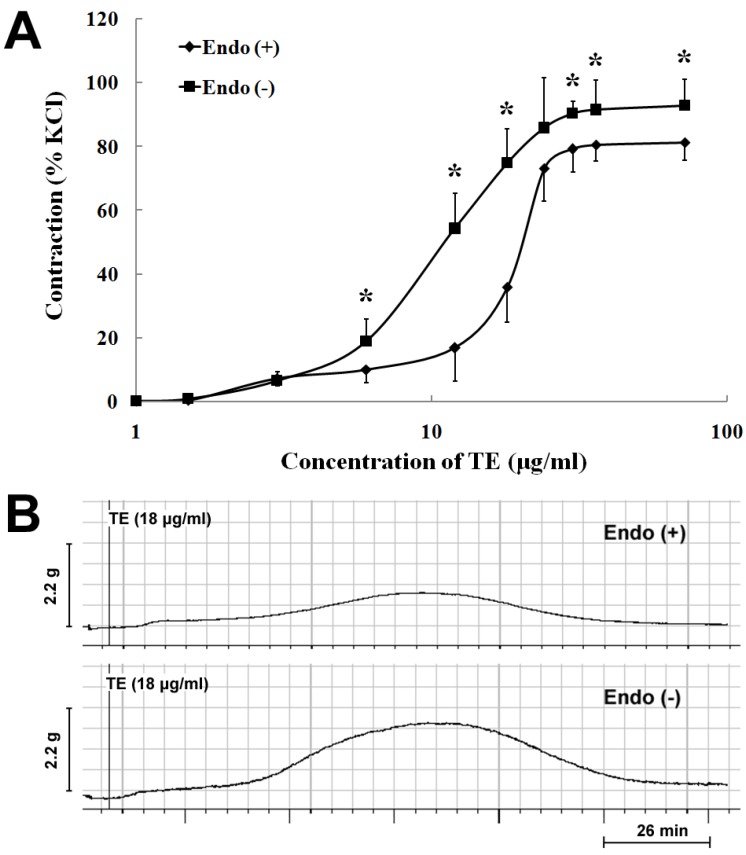
Vasoconstrictor responses induced by the tentacle extract (TE) from the jellyfish, *C. capillata*, *in vitro*. (**A**) TE (1.5–72 µg/mL) induced the concentration-contraction response curve in the endothelium-intact and -denuded aorta rings; (**B**) the representative trace demonstrating the contractile responses of TE (18 μg/mL) in the endothelium-intact and -denuded aorta rings. Data are shown as the mean ± SD and expressed as the percentage of the maximum KCl contraction. (*n* = 7). Endo (+): endothelium-intact; Endo (−): endothelium-denuded. * *p* *<* 0.05 *vs*. Endo (+).

In the endothelium-intact rings, TE (1.5–72 μg/mL) produced a concentration-dependent increase in contraction with an EC_50_ of 18.25 ± 0.13 µg/mL (*n* = 7; Figure 1A; Table 1). Endothelium denudation caused concentration-dependent leftward shifts of the TE concentration-contraction response curve (EC_50_ = 11.93 ± 0.09 μg/mL), with a significant increase in the maximum response (92.91 ± 8.15% *vs.* 81.33 ± 5.56% of 60 mM KCl) (*n* = 7; Figure 1A; Table 1). Besides, TE (18 μg/mL) produced a bell-shaped contractile response curve in both endothelium-intact and -denuded aortas (Figure 1B), which peaked around 60 min and, then, decreased, but not completely returning to the baseline (Figure 1B). Nevertheless, the amplitude of contraction in the endothelium-denuded aorta was stronger than that in the endothelium-intact one (Figure 1A,B).

**Table 1:** Effects of various pretreatments on TE-induced concentration-contraction response curves in isolated aortic rings.

Pretreatments	Endothelium	EC_50_ (ug/mL)	*E*_max_ (% KCl)	*n*
No drug	+	18.25 ± 0.13	81.33 ± 5.56	7
l-NAME(1 μM)	+	12.13 ± 0.12 *	97.80 ± 9.81 *	7
Indomethacin (1 μM)	+	18.42 ± 0.14	76.21 ± 5.64	7
No drug	−	11.93 ± 0.09 *	92.91 ± 8.15 *	7
Low-calcium	−	18.74 ± 0.47 ^#^	33.59± 3.62 ^#^	6
Calcium-free	−	25.89 ± 6.14 ^#^	4.97 ± 3.54 ^#^	6
Diltiazem (1 μM)	−	18.65 ± 0.36 ^#^	40.17 ± 7.27 ^#^	6
Nifedipine (1 μM)	−	18.52 ± 0.27 ^#^	55.67 ± 5.22 ^#^	6
Verapamil (1 μM)	−	18.72 ± 0.39 ^#^	49.25 ± 5.16 ^#^	6
2-APB (10 μM)	−	12.59 ± 0.32	55.30 ± 11.92 ^#^	6
Heparin (2500 IU)	−	36.03 ± 0.18 ^#^	49.57 ± 5.63 ^#^	6
PKC 412 (5 μM)	−	12.15 ± 0.12	85.94 ± 13.93	6
U-73122 (10 μM)	−	12.11 ± 0.19	82.70 ± 10.47	6
Propranolol (1 μM)	−	12.15 ± 0.12	89.59 ± 10.71	6
Phentolamine (1 μM)	−	11.81 ± 0.07	94.49 ± 13.02	6

TE, tentacle extract; l-NAME, *N*^ω^-nitro-l-arginine methyl ester; 2-APB, 2-aminoethoxydiphenyl borate; EC_50_, the concentration (μg/mL) required to produce 50% maximum response; *n*, number of arteries (each taken from separate rats). * *p* < 0.05 *vs.* no drug with endothelium; ^#^ *p* < 0.05 *vs.* no drug without endothelium; one-way ANOVA with Dunnett’s posttest for multiple comparisons.

**Figure 2:**
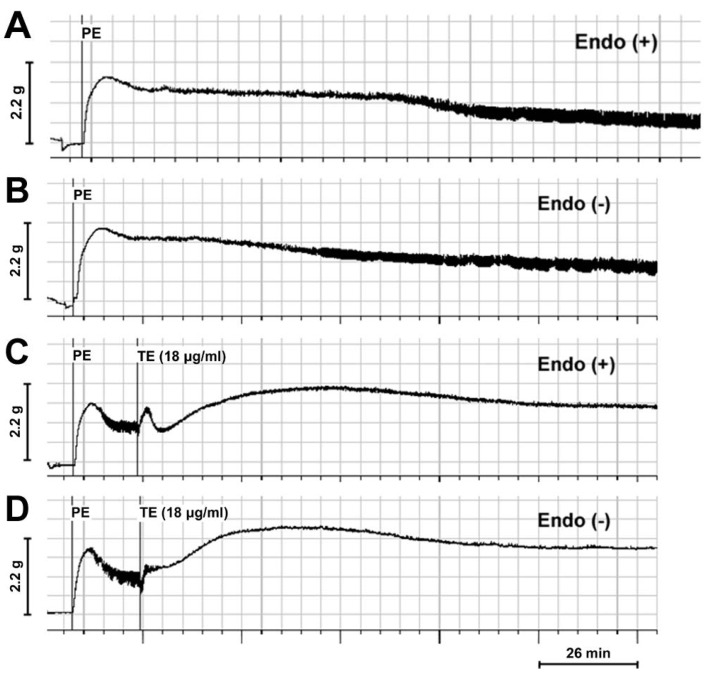
Trace demonstrating the contractile responses of phenylephrine (PE) (1 μM) pre-contracted rings in the absence and presence of TE from the jellyfish, *C. capillata* (18 μg/mL). (**A**) PE pre-contracted endothelium-intact rings in the absence of TE; (**B**) PE pre-contracted endothelium-denuded rings in the absence of TE; (**C**) PE pre-contracted endothelium-intact rings in the presence of TE; (**D**) PE pre-contracted endothelium-denuded rings in the presence of TE. Endo (+): endothelium-intact; Endo (−): endothelium-denuded.

In the PE (1 μM) pre-contracted rings, TE (18 μg/mL) significantly enhanced PE-induced contraction and increased PE-induced maximum contraction in both endothelium-intact (Figure 2A,C) and -denuded aortas (Figure 2B,D). Interestingly, a three-phase response pattern was induced by TE in endothelium-intact aorta with an initial contraction, followed by transient relaxation and sustained contraction (Figure 2C), while the brief relaxation responses seemed to be abolished, and only a sustained contraction was observed after the removal of endothelium (Figure 2D).

### 3.2. Effects of Endothelium-Derived Vasodilators on TE-Induced Contraction in the Endothelium-Intact Aorta

**Figure 3:**
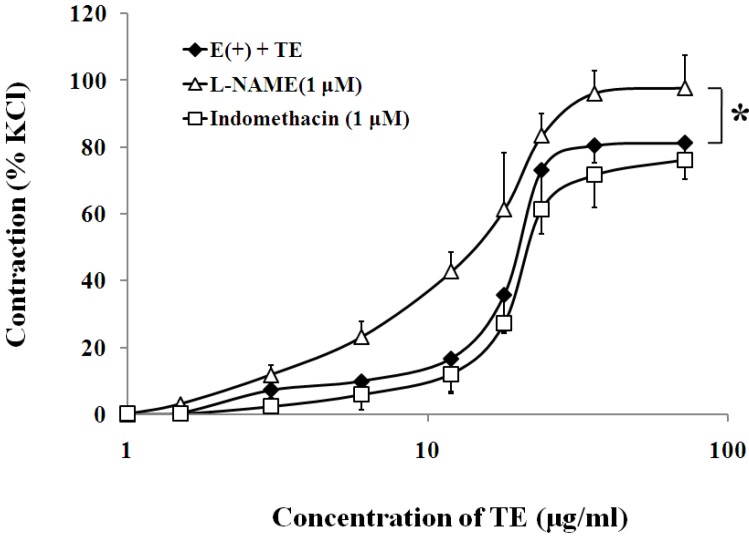
Effects of l-NAME (Δ) or indomethacin (□) on TE-induced (1.5–72 μg/mL) concentration-contraction response curve in the endothelium-intact aorta rings. Data are shown as the mean ± SD and expressed as the percentage of the maximum KCl contraction (*n* = 7). E (+): endothelium-intact. * *p <* 0.05 *vs.* E (+) + TE.

In the endothelium-intact aorta, nitric oxide synthase (NOS) inhibitor, l-NAME (1 μM), significantly increased the sensitivity (EC_50_ of 12.13 ± 0.12 *vs*. 18.25 ± 0.13 μg/mL; *n* = 6; Figure 3; Table 1) and maximum contraction to TE (97.80 ± 9.81% *vs*. 81.33 ± 5.56% of 60 mM KCl; *n* = 6; Figure 3; Table 1), while cyclooxygenase inhibitor, indomethacin (1 μM), had no effect on the sensitivity or maximum response to TE (Figure 3; Table 1).

### 3.3. Effects of Calcium Channel Blockers on TE-Induced Contraction in the Endothelium-Denuded Aorta

**Figure 4:**
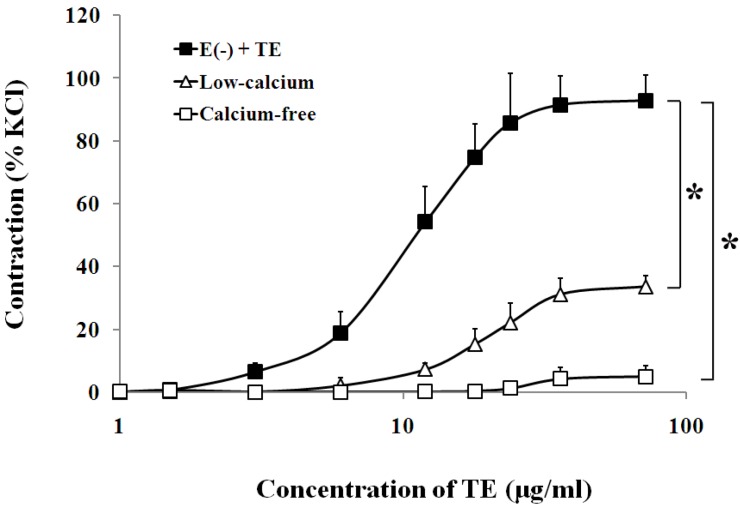
Effects of the low-calcium Krebs solution (Δ) or calcium-free Krebs solution (□) on TE-induced (1.5–72 μg/mL) concentration-contraction response curve in the endothelium-denuded aorta rings. Data are shown as the mean ± SD and expressed as the percentage of the maximum KCl contraction. (*n* = 6). E (−): endothelium-denuded. * *p <* 0.05 *vs.* E (−) + TE.

**Figure 5:**
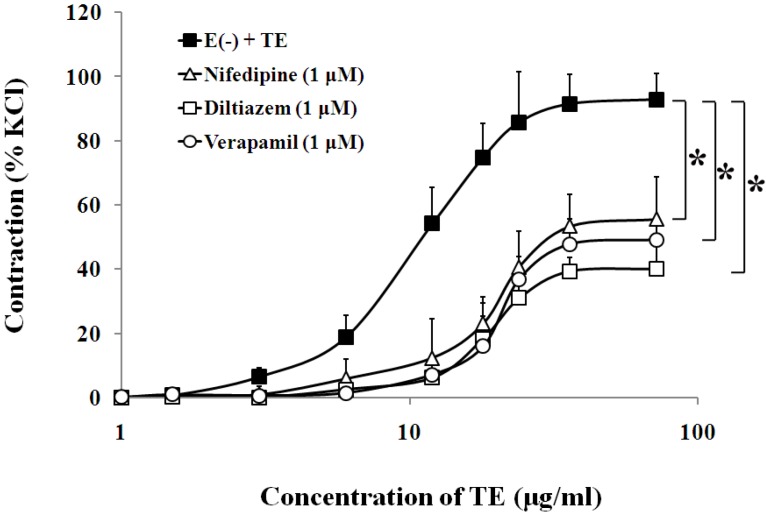
Effects of nifedipine (Δ), diltiazem (□) or verapamil (○) on the TE-induced (1.5–72 μg/mL) concentration-contraction response curve in the endothelium-denuded aorta rings. Data are shown as the mean ± SD and expressed as the percentage of the maximum KCl contraction. (*n* = 6). E (−): endothelium-denuded. *p < 0.05 *vs.* E (−) + TE.

**Figure 6:**
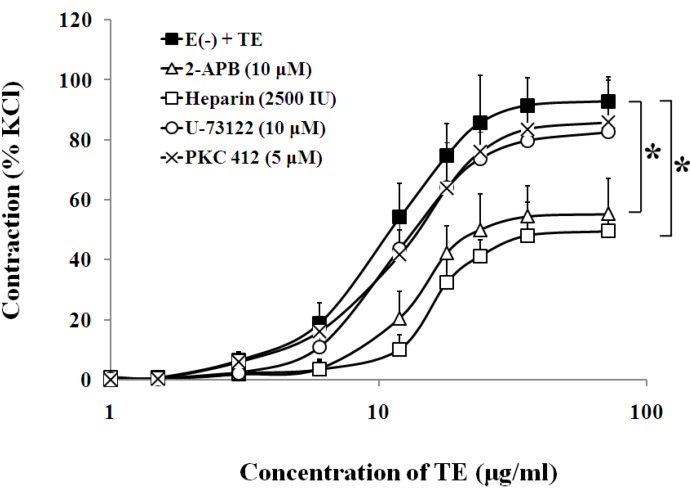
Effects of 2-APB (Δ), heparin (□) U-73122 (○) or PKC 412 (×) on the TE-induced (1.5–72 μg/mL) concentration-contraction response curve in the endothelium-denuded aorta rings. Data are shown as the mean ± SD and expressed as the percentage of the maximum KCl contraction. (*n* = 6). E (−): endothelium-denuded. * *p <* 0.05 *vs.* E (−) + TE.

In the endothelium-denuded aorta, calcium-free Krebs solution, low-calcium (1 mM CaCl_2_) solution (Figure 4, Table 1), VOCC blockers, including nifedipine (1 μM), diltiazem (1 μM) and verapamil (1 μM) (Figure 5, Table 1), and IP_3_ receptor blockers, including 2-APB (10 μM) and heparin (2500 IU) (Figure 6, Table 1) all caused rightward shifts of the TE concentration-response curve, with a significant decrease in the TE-induced maximum contraction (Figure 6, Table 1). Nevertheless, neither phospholipase C inhibitor (U-73122, 10 μM) nor PKC inhibitor (PKC 412, 5 μM) caused a change in sensitivity or maximum response to TE (Figure 6, Table 1).

### 3.4. Effects of Sympathetic Nerve System on TE-Induced Contraction in the Endothelium-Denuded Aorta

**Figure 7:**
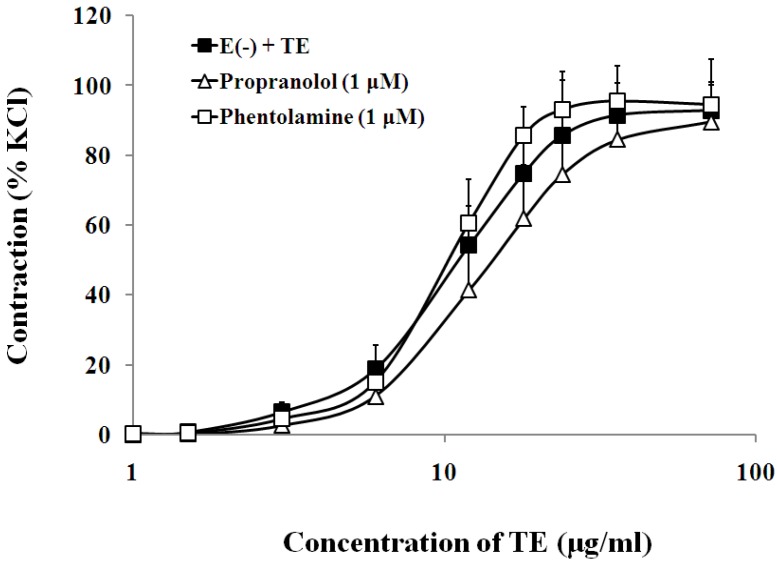
Effects of propranolol (Δ) or phentolamine (□) on the TE-induced (1.5–72 μg/mL) concentration-contraction response curve in the endothelium-denuded aorta rings. Data are shown as the mean ± SD and expressed as the percentage of the maximum KCl contraction (*n* = 6). E (−): endothelium- denuded. * *p <* 0.05 *vs.* E (−) + TE.

The presence of either β-adrenoceptor antagonist, propranolol (1 μM), or α-adrenoceptor antagonist, phentolamine (1 μM), had no significant effect on the sensitivity or maximum response to TE in the endothelium-denuded aorta (Figure 7, Table 1).

## 4. Discussion

Clinical reports and animal studies suggest that death caused by jellyfish envenomation is attributed primarily to the cardiovascular toxicity of the venom [4,8]. Consequently, much research has been conducted in regard to pharmacological and cardiovascular characterization of jellyfish venom [23,24,25,26]. However, the wide variation in venom extraction techniques has resulted in varying pharmacological activities from different extracts and has made it almost impossible to compare the similar research performed to date [7,8]. Fortunately, a superior technique for the separation of nematocyst venom (NV) with the tentacle extract (free of the nematocysts) [27,28] has been developed, which represents a technical advancement of jellyfish venom isolation. After that, researchers have shown a greater concern with the nematocyst venom rather than the tentacle extract [5,7]. However, there has been evidence demonstrating that the bioactive proteins with cardiovascular toxicity in both jellyfish NV and TE are probably encoded by the same gene fragment [6,29,30]. Besides, Ramasamy [23,24] found that both the NV and TE from *Chironex fleckeri* and *Chiropsella bronzie* could produce a pressor response in the first few minutes. Based on these findings, we have also evaluated the cardiovascular effects of the NV and TE from *C. capillata*, and the result showed that NV and TE share similar cardiovascular activity *in vivo* [6]. Besides, using the Langendorff-perfused rat heart model, we have further verified that TE also possesses direct cardiac toxicity [9]. Given the problem of an insufficient venom source, we proposed that TE may provide a potential alternative to NV, with a much richer source for the acquisition of cardiovascular toxicity proteins [6]. Therefore, herein, using TE as a toxin example, we examine the vascular activity of TE from the jellyfish, *C. capillata*, for the first time.

Using isolated rat aortic rings, we confirmed that TE could induce concentration-dependent contractile responses in both endothelium-intact and -denuded aortas, which is consistent with the results on *C. fleckeri* [23], *C. bronzie* [24] and *Malo maxima* [31], indicating that TE could contract VSMC directly. Interestingly, the amplitude of contraction in the endothelium-denuded aorta was much stronger than that in the endothelium-intact aorta, implying that TE may also mediate the release of endothelium-dependent vasodilators. This hypothesis was further supported by the tests in PE (1 μM) pre-contracted rings, where a three-phase response was characterized with an initial contraction, followed by transient relaxation and sustained contraction in endothelium-intact aorta, while the brief relaxation response was abolished after endothelium denudation. In addition, the following drug intervention experiments found that the inhibition of eNOS by l-NAME significantly increased the sensitivity and maximum contraction to TE, while the inhibition of cyclooxygenase by indomethacin had no effect. These results suggested that TE may also cause a weak functional relaxation in addition to vasoconstriction, which may be mediated by nitric oxide. Thus, we speculated that TE-induced contractile response is the sum of both the contractile and relaxant actions of the venom. In fact, the dual contractile/relaxant properties may be a characteristic of certain venoms from venomous animals, including *C. fleckeri* [32], *C. barnesi* (also known as Irukandji jellyfish) [33], as well as some scorpions [34] and spiders [35]. Therefore, the next pharmacologic study was performed only in the endothelium-denuded aorta.

It has been well established that changes in [Ca^2+^]_c_ are the main events that regulate the contractile state of VSMC. Furthermore, usually, an increase in [Ca^2+^]_c_ results in VSM contraction, and a decrease in [Ca^2+^]_c_ results in VSM relaxation [18]. In response to vasoconstrictor stimuli, an increase in [Ca^2+^]_c_ may arise from the opening of calcium channels in the plasma membrane, allowing the influx of extracellular Ca^2+^, or from the release of Ca^2+^ stores, such as SR [36]. To clarify the dependence of TE-induced contraction on extracellular calcium, we tested the effects of TE on the contractile force of the aortic rings in standard Krebs solution, low-calcium Krebs solution (1 mM CaCl_2_) and calcium-free Krebs solution, respectively. The results showed that calcium-free Krebs solution nearly abolished TE-induced contraction and low calcium concentration (1 mM) significantly attenuated the sensitivity and maximum contraction to TE, suggesting that contractile responses induced by TE appeared to be primarily dependent on extracellular calcium concentrations. It has also been demonstrated that transmembrane Ca^2+^ influx depends on the classical ionotropic function of ion channels, among which the l-type VOCCs are particularly relevant. In response to membrane depolarization, VOCC opening permits the influx of Ca^2+^ from the extracellular space. This subsequently activates the Ca^2+^-induced Ca^2+^-release (CICR) mechanism via ryanodine receptor (RyR) activation and causes a myogenic contraction [37,38]. Thus, we used three different types of l-type calcium channels blockers, such as dihydropyridines (e.g., nifedipine), benzothiazepines (e.g., diltiazem) and phenylalkylamines (e.g., verapamil), to explore if TE-induced contractile response was associated with the l-type Ca^2+^ channel on plasma membrane. Our results showed that these drugs were effective in shifting the TE concentration-contraction response curve to the right and dramatically attenuating TE-induced maximum contraction, which was also consistent with another author’s study [39], in which *C.* *fleckeri* venom*-*induced contractile response was significantly reduced by felodipine, an antagonist selective for peripheral l-type Ca^2+^ channels. Therefore, we hypothesized that TE may affect VOCCs, trigger the CICR mechanism, disturb Ca^2+^ signaling systems, lead to Ca^2+^ overload and, finally, cause vasoconstriction [38]. In addition, another important mechanism of aortic contraction may arise from the release of Ca^2+^ from the SR. This pathway involves stimulation of phospholipase C to produce 1,2-diacylglycerol (DAG) and IP_3_. Subsequently, DAG activates the myosin light chain through activation of PKC, and IP_3_ induces Ca^2+^ release from the SR by opening IP_3_ receptors [36]. Our results showed that TE-induced contraction seemed unaffected by antagonists of phospholipase C and PKC, which suggested that the vascular effects of TE are not due to the activation of the PLC/DAG/PKC pathway. On the other hand, the TE concentration-response curve was shifted to the right, and the maximum contraction was markedly decreased by IP_3_ receptor blockers, including 2-APB and heparin, which implied that IP_3_R was activated by TE, leading to SR Ca^2+^ release and an increase in [Ca^2+^]_c_.

It has been reported that “Irukandji syndrome”, a medical condition arising from carybdeid jellyfish envenoming, may be attributed to excessive catecholamines (CAs) [40]. Moreover, hypertension due to CAs has been demonstrated in the animal model of *Carukia barnesi* envenomation [24]. As is well known, excited sympathetic nerve endings could release excessive CAs, causing small artery and vein contraction and cardiac output increase, finally leading to hypertension [20]. It does so, in a large part, by activating both α- and β-adrenergic receptors (ARs) on VSMCs and cardiomyocytes. Thus, we pre-incubated the aortic rings with propranolol (1 μM), a nonselective and competitive blocker for β-ARs, and phentolamine, a non-selective α-ARs antagonist, before TE administration. The results showed that the contraction of aortas was insensitive to antagonists of α- and β-adrenoceptors, indicating that the vasoconstrictive effects of TE are not due to the activation of sympathetic nerves, which is also consistent with previous studies on *C. fleckeri* venom by Hughes *et al.* [32] and Winter *et al.* [39].

## 5. Conclusions

In conclusion, a direct vasoconstrictive effect of TE from the jellyfish, *C. capillata*, was verified in isolated aorta rings. Besides, TE may also cause a weak functional relaxation, which may be mediated by endothelium-derived vasodilator (nitric oxide). The major pharmacologic findings in isolated endothelium-denuded rat aorta are as follows: (1) TE-induced contraction is primarily dependent on calcium influx via VOCCs from the extracellular space; (2) TE-induced contraction is also associated with the activation of IP_3_R, leading to SR Ca^2+^ release and an increase in [Ca^2+^]_c_; (3) the vasoconstrictive effects of TE are not due to the activation of the PLC/DAG/PKC pathway; and (4), finally, the sympathetic nerve system is not involved in TE-induced vasoconstriction.
